# Vitamin C in the Treatment of Colorectal Cancer: Between Hope and Despair

**DOI:** 10.3390/cancers18040654

**Published:** 2026-02-17

**Authors:** Mathias Wasmer, Markus Weber, Seraina Faes

**Affiliations:** City Hospital Zurich, Department of Visceral, Thoracic and Vascular Surgery, Birmensdorferstrasse 497, 8036 Zurich, Switzerland; mathias.wasmer@stadtspital.ch (M.W.); markus.weber@stadtspital.ch (M.W.)

**Keywords:** cancer, colorectal, vitamin C, ascorbate

## Abstract

Despite recent progress in different treatment modalities, colorectal cancer remains a leading cause of cancer-related death, highlighting the need to further develop novel treatment strategies. In this context, over recent decades, several experimental studies have demonstrated that high doses of vitamin C provide anti-cancer benefits in various colorectal cancer models. Intravenous administrations of vitamin C are necessary to reach these high concentrations in tumors. Tumors harboring *KRAS* or *BRAF* mutations or driven by the HIF1α signaling pathway are particularly sensitive to high-dose vitamin C. In addition, high doses of vitamin C increase the efficacy of other treatments when used in combination, including chemotherapies, targeted therapies, and immunotherapies. Whilst results of experimental studies were promising, their translation into clinical protocols has been disappointing. So far, very few clinical data support the anti-cancer benefits of vitamin C in colorectal cancer patients. This lack of success highlights the need to ameliorate the selection of patients based on biomarkers and to refine treatment protocols to achieve high-dose vitamin C in tumors. In this review, we analyze in vitro and in vivo studies that investigated the effect of vitamin C in colorectal cancer cells and point out the anti-cancer mechanisms of vitamin C. We further examine clinical trials that tested vitamin C in colorectal cancer patients and address several points that still need to be investigated in order to fully define the role of vitamin C in the treatment of colorectal cancer.

## Simple Summary

Vitamin C displays anti-cancer properties in part via its pro-oxidative activity. Whereas most evidence has been obtained in preclinical studies, recent clinical trials suggest that vitamin C might provide anti-cancer benefits in cancer patients. In this review, we summarize the available evidence for a role of vitamin C in the treatment of colorectal cancer. Importantly, understanding the anti-cancer mechanisms of vitamin C could help design pertinent treatment protocols for colorectal cancer patients.

## 1. Introduction

### 1.1. Colorectal Cancer

Colorectal cancer (CRC) remains a significant health challenge, being one of the most frequent types of cancer and representing a leading cause of cancer-related deaths [1]. In 2022, over 1.9 million new cases of CRC and 904 000 CRC-related deaths were estimated worldwide [2]. Of concern, the global incidence of CRC is expected to increase with a rise of 60–70% in the total number of deaths due to CRC by the year 2035 [3,4]. Whilst the majority of new diagnoses and deaths due to CRC still occur in adults over 70 years of age, younger adults are increasingly affected by this disease, which led to the definition of early-onset CRC if diagnosed before the age of 50 [5,6,7]. Incidence and mortality of CRC exhibit marked disparities with age, geographical region, and median human development index [1,6].

Several factors have been identified to contribute to CRC development, including smoking, sedentary behavior, obesity, high consumption of red and processed meat, low intake of dietary fiber, inflammatory bowel diseases, and alcohol consumption [8,9,10,11,12]. A combination of these modifiable behavioral lifestyle factors can explain an up to 3.89-fold risk increase for CRC development [13]. Gut microbiome and epigenetic changes due to dietary factors are further suspected to influence CRC development [14,15]. In addition, genetic factors increase the risk of CRC development as observed in patients with family history of CRC, familial adenomatous polyposis, and Lynch syndrome [16,17,18]. Protective factors have also been characterized, such as physical activity, the use of non-steroidal anti-inflammatory drugs, and antioxidant agents.

CRC develops via several distinct pathways [19,20]. Different mutations in genes resulting in up- and downregulation of specific tumor suppressor genes or oncogenes or mediators involved in regulation of the cell cycle, cell proliferation, or apoptosis, as well as regulation of cell interactions, are only a few to be mentioned. Alterations of methylation patterns also play an important role in the onset of CRC by dysregulation of different mechanisms on a cellular level.

At diagnosis, approximately 20% of CRC cases already present distant metastasis [21]. The 5-year survival rate of this metastatic form of the disease reaches approximately 15%. In contrast, stage 1 CRC is associated with a good prognosis, highlighting the importance of detecting the disease early. Multiple screening tests are available to detect colon cancer, including endoscopic tests (colonoscopy, sigmoidoscopy), stool-based tests (fecal immunochemical tests, guaiac-based fecal occult blood tests, multitarget stool DNA tests), and radiological procedures (computed tomography colonography).

Treatment of metastatic CRC remains difficult and is in constant development. Nevertheless, several targeted therapies in combination with chemotherapy have been associated with increased overall survival in metastatic CRC. These include anti-angiogenic agents (bevacizumab, ramucirumab, aflibercept, regorafenib, fruquintinib) [22,23,24,25], anti-EGFR treatments (cetuximab, panitumumab), immunotherapies targeting checkpoint inhibitors (nivolumab, pembrolizumab, ipilimumab) [26], anti-HER-2 therapies (trastuzumab, pertuzumab, lapatinib) [27,28], *BRAF* inhibitors (encorafenib) [29], and *KRAS* G12C inhibitors (sotorasib, adagrasib) [30]. Interestingly, gene expression analysis helps in choosing the appropriate treatment. As first-line treatments, metastatic CRC harboring mismatch-repair-deficient enzymes and microsatellite instability is susceptible to immune checkpoint inhibitors. In CRC with microsatellite stability, treatment will be dictated by *RAS* mutational status and the location of the tumor. Left-sided tumors with wild-type *RAS* will benefit from anti-EGFR treatments in combination with chemotherapy. Right-sided tumors with wild-type *RAS* or tumors with mutated *RAS* will be treated with anti-VEGF agent and chemotherapy. Nevertheless, despite major progress in its management, CRC is a leading cause of cancer-related death, highlighting the need to further explore additional treatment strategies.

### 1.2. Vitamin C

**Figure 1:**
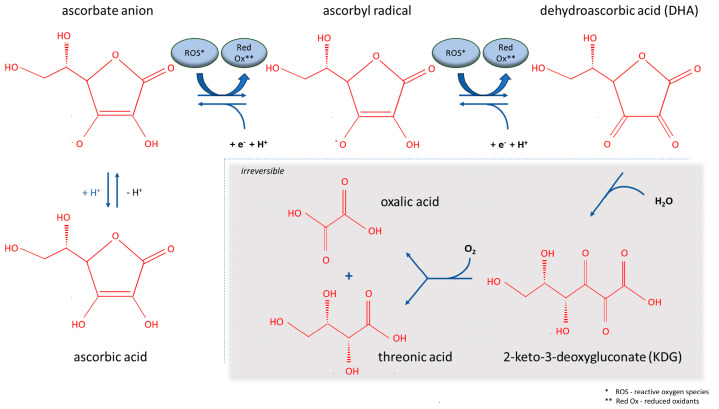
Molecular formula and redox reaction of vitamin C.

Vitamin C, or ascorbic acid or ascorbate, is an essential nutrient found in fruits and vegetables. Unlike most animals, humans are not able to synthesize vitamin C due to inactivating mutations in the gene of L-gulonolactone oxidase, which is involved in the last step of vitamin C synthesis [31]. Under physiological conditions, vitamin C is mostly present in the form of ascorbate anion. Since ascorbate anion can donate two electrons, it exists in different redox forms. Loss of one electron leads to the generation of the ascorbate radical, which is further oxidized into dehydroascorbic acid (DHA) after the loss of a second electron (Figure 1) [32].

DHA either enters the cell through glucose transporters (GLUTs), mainly GLUT1 and GLUT3, or becomes metabolized into 2,3-L-diketogluconate, which will be degraded into oxalic and threonic acids and eliminated by the kidneys. Hence, with its electron donor property, vitamin C can reduce reactive oxygen species (ROS) and act as an antioxidant. The antioxidant properties of vitamin C are however not limited to its function as a free radical scavenger but further involve interaction with the antioxidant cellular systems such as thioredoxin and glutathione, stimulation of antioxidative enzymes, including catalase and superoxide dismutase, and enhancing the activity of transcription factors like Nrf2 that regulate the antioxidant gene transcription responses [33]. On the contrary, vitamin C can also act as a pro-oxidant by different mechanisms. In fact, vitamin C can reduce metals, in particular iron, thereby maintaining a pool of ferrous iron that will be oxidized by oxygen, generating ROS via the Fenton reaction. This process occurs extracellularly as well as intracellularly following entry of vitamin C into cells via sodium-dependent vitamin C transporters (SCVTs). In addition, vitamin C reduces intracellular levels of the antioxidant glutathione, therefore increasing intracellular oxidative stress. This happens when DHA enters cells and is reduced back to vitamin C by consuming reduced glutathione. Therefore, vitamin C as an electron donor can act either as a anti- or pro-oxidant depending on where the electrons go [31].

**Figure 2:**
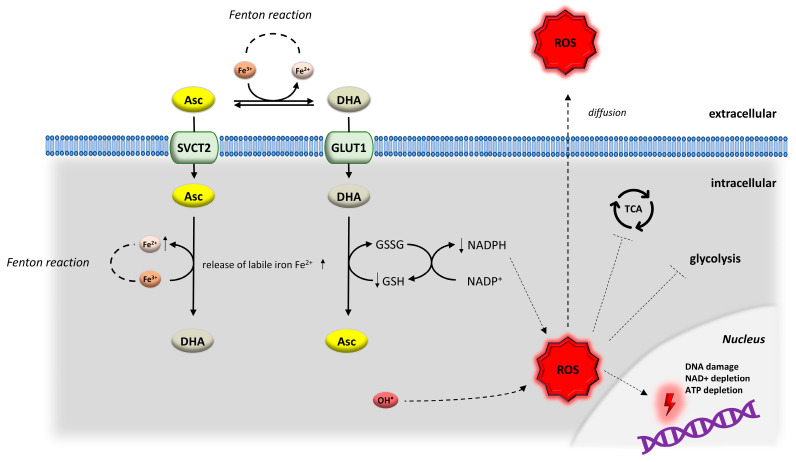
Anti- and pro-oxidant mechanisms of vitamin C. Asc: ascorbic acid, DHA: dehydroascorbic acid, GLUT: glucose transporter, SVCT: sodium-dependent vitamin C transporter, Fe^2+^/Fe^3+^: ferrous/ferric ions, ROS: reactive oxygen species, GSH/GSSG: reduced glutathion/glutathione disulfide, DNA: deoxyribonucleic acid, NADH/NAD+: nicotinamide adenine dinucleotide, redox forms, ATP: adenosine triphosphate. Fenton reaction: Generation of highly reactive hydroxyl radicals by reaction of ferrous iron with hydrogen peroxide.

Besides its redox functions, vitamin C influences several physiologic processes, including collagen, norepinephrine and carnitine synthesis, HIF1α degradation, tyrosine catabolism, and DNA demethylation [34]. Anti- and pro-oxidant mechanisms of vitamin C are summarized in Figure 2.

Several in vitro, in vivo and clinical studies have shown that high doses of vitamin C display anti-tumor activity without affecting normal cells [35,36,37,38]. Several mechanisms have been identified that are responsible for this effect [39]. These involve increasing oxidative stress, epigenetic reprogramming, inhibition of growth promoting kinases, inhibition of the HIF1α signaling pathway, and immune modulatory effects [31,37]. In addition, it was further demonstrated that vitamin C potentiates the anti-cancer effects of different cancer treatment modalities including chemotherapy, radiotherapy and immunotherapy. In this context, emerging evidence shows that CRC patients whose tumors harbor *KRAS* or *BRAF* mutations might particularly benefit from vitamin C treatment. Here, we give an overview of the experimental studies that investigated the effect of vitamin C in CRC cells and detail the responsible anti-cancer mechanisms. We further discuss the clinical trials that tested vitamin C administration in CRC patients.

## 2. Experimental Evidence for an Anti-Cancer Effect of Vitamin C in CRC

### 2.1. Experimental Evidence of Vitamin C In Vitro and In Vivo

Several studies have demonstrated that vitamin C displays anti-cancer activities against CRC cell lines both in vitro and in vivo.

Using isogenic human colon cancer cell lines with either mutant or wild-type alleles of *KRAS* and *BRAF*, it was observed that vitamin C decreased mutant cell growth and colony formation compared to wild-type counterparts [40]. High doses of vitamin C also reduced the growth of tumor xenografts established with *KRAS* or *BRAF* mutant colon cancer cell lines. Administration of the antioxidant N-acetylcysteine (NAC) prevented the anti-cancer effects of vitamin C, supporting that increased ROS were responsible for this effect. Similarly, in a transgenic mouse model where intestinal tumors are driven by *Apc* mutation or combined *Apc/Kras* mutations, vitamin C reduced the number of polyps in *Apc/Kras*-mutated mice but not in *Apc*-mutated mice. Molecular analysis revealed that, in *KRAS*- or *BRAF*-mutated colon cancer cells, the uptake of the oxidized form of vitamin C, DHA, was augmented via the GLUT1 transporter. Consequently, oxidative stress was increased, as DHA is converted back to vitamin C in cancer cells at the expense of the antioxidative machinery. In turn, augmented ROS led to the direct inactivation of GAPDH as well as an indirect inactivation of GAPDH via the activation of poly (ADP-ribose) polymerase, resulting in depletion of nicotinamide adenine dinucleotide (NAD), a cofactor of GAPDH. As *KRAS*- or *BRAF*-mutated cells are highly glycolytic, inhibition of GAPDH results in ATP depletion and cancer cell death. Hence the selective effects of vitamin C on *KRAS*- or *BRAF*-mutated colon cancer cells are caused by *KRAS* or *BRAF* mutants’ mediated high-level expression of GLUT1 in combination with glycolytic addiction.

Vitamin-C-induced metabolic alteration of the glycolysis pathway and increased oxidative stress in CRC were further confirmed in other studies. For instance, relatively high doses of vitamin C were needed to decrease HT29 cell growth [41]. Generation of hydrogen peroxide (H_2_O_2_) was presumably responsible for the anti-cancer effects, since NAC and reduced glutathione abolished the cytotoxic effects of vitamin C. Metabolomic analysis of HT29 cells treated with cytotoxic concentrations of vitamin C revealed that the energy flux in glycolysis and the tricarboxylic acid (TCA) cycle was disrupted, resulting in reduced ATP production. In particular, conversion of glyceraldehyde 3-phosphate to D-glycerate 1,3-bisphosphate, which is mediated by glyceraldehyde 3-phosphate dehydrogenase in the presence of NAD, was suppressed. Additional analysis showed that vitamin C reduced the levels of NAD in HT29 cells, and addition of NAD prevented vitamin-C-mediated HT29 cell death. Moreover, the reduced glutathione/oxidized glutathione ratio was decreased, confirming increased cellular oxidative stress induced by vitamin C. Liquid chromatography tandem mass spectrometry analysis confirmed disruption of glycolysis by high-dose vitamin C with increased compensatory pentose phosphate shunt in HT-29 cells [42].

Consistent with these findings, vitamin C decreased glucose consumption, lactate and ATP production in HCT116 cells [43]. This led to reduced HCT116 cancer cell viability and HCT116 tumor xenograft growth. Similarly, vitamin C downregulated glycolysis and led to an energy crisis in LoVo and SW480 cells [44]. This was a consequence of MAPK pathway inhibition that resulted in decreased GLUT1 and PKM2 expression. Of note, here and in contrast to previously mentioned studies, the anti-cancer activity of vitamin C was due to reduced intracellular ROS levels, which induced the translocation of Ras from the plasma membrane into the cytosol and the inhibition of the Ras/MEK/Erk signaling pathway. Finally, vitamin C also affected ATP production in DLD-1 and SW480 cells [45]. Here, vitamin C treatment led to decreased HIF1α stability and activity. Consequently, Glut1 and PDK1 expression were downregulated by vitamin C in conditions that mimic hypoxia. Reduced Glut1 and PDK1 expression by vitamin C was further confirmed in SW480 tumor xenografts.

Besides the effects of vitamin C in *KRAS/BRAF*-mutated CRC, other studies have demonstrated that the anti-cancer potential of vitamin C might be particularly efficient in tumors driven by the HIF1α signaling pathway [46]. In fact, ascorbate is a cofactor to HIF hydroxylases that in physiological conditions induce HIF1α hydroxylation, resulting in its proteasomal degradation. Hence, in the absence of vitamin C, HIF1α is not degraded and the HIF signaling pathway is activated. In the context of CRC, ascorbate significantly reduced HIF1α protein expression in WiDr colon cancer cells following stimulation with various HIF1 inducers, including CoCl_2_, DFO, DMOG or 5% O_2_ [47]. Also, overexpression of a constitutively active HIF1α subunit by lentiviruses in HCT116 colon cancer cells increased HCT116 death mediated by vitamin C [48]. Activation of the HIF pathway via treatment with the chemical DMOG provided similar effects. Contrasting with the in vitro experiments, high-dose vitamin C did not demonstrate anti-cancer effects in HCT116 tumor xenografts that overexpressed active forms of HIF1α.

Genetic determinants that influence the efficacy of vitamin C have further been supported by the observation that intact p53 in cancer cells increases the anti-tumor activity of vitamin C [49]. Indeed, viability of HCT116 colon cancer cells that express wild-type p53 was significantly reduced compared to HCT116 cells that do not express p53 when treated with vitamin C. One proposed mechanism responsible for this effect was that p53 increases the production of H_2_O_2_ mediated by vitamin C. In addition, vitamin C favored a pro-oxidant transcriptional network in p53-positive but not in p53-negative colon cancer cells. Similar observations were made in vivo, where vitamin C decreased the growth of HCT116 p53-positive tumor xenografts but had no effect on p53-null HCT116 tumors.

Additional evidence for an anti-cancer potential of vitamin C in CRC was found. Exposure for one hour of cancer cell lines and human normal cells to ascorbic acid concentrations that correspond to plasma ascorbate levels found in humans following iv administration selectively reduced cancer cell survival and had no effect on normal cells. Among the cell lines tested, the mouse colon cancer cell line CT26 was one of the most sensitive with an IC_50_ value of less than 5 mM. In contrast, HT29 IC_50_ was estimated to be higher than 20 mM. In addition, colony growth of the human colon cancer cell line HT29 was not affected by vitamin C [50,51].

Treatment of LS174T, HT29, and SW480 CRC cells with high-dose vitamin C suppressed cell proliferation and induced cell apoptosis [52]. High-dose but not low-dose injections of vitamin C decreased the growth of LS174T tumor xenografts. CD31 staining of xenografts revealed that vitamin C decreased tumor angiogenesis. In addition, vitamin C also reduced metastasis formation in an intraperitoneal implantation metastasis model. Downregulation of the long non-coding RNA MALAT1, which promotes tumor growth and metastasis, was associated with vitamin C treatment.

The anti-cancer efficacy of vitamin C was also tested in CT26 tumor allografts [53]. Two sets of similar experiments were performed and compared the weight of CT26 tumors grown in untreated mice or mice treated with vitamin C. While vitamin C significantly reduced the weight of CT26 tumors in the first set of experiments, it had no significant effect in the second one. The anti-cancer efficacy of vitamin C in CT26 tumor allografts was also observed when vitamin C was delivered intratumorally [54]. Vitamin-C-induced anti-cancer effects were also demonstrated in HT29 cells as well as HT29 tumor xenografts [55]. In vitro, treatment of HT29 cells with vitamin C resulted in cancer cell necrosis and decreased cell growth. In vivo, treatment of mice bearing HT29 xenografts with high-dose vitamin C but not with lower doses significantly reduced tumor growth and increased mouse survival. In addition, peritoneal metastatic spreading was found in untreated animals or animals treated with lower doses of vitamin C but not in mice receiving the highest doses. Of note, mRNA of tRNA synthetases and translation initiation factor subunits were downregulated in tumors following high-dose treatment of vitamin C. Consistent with these findings, another study revealed that vitamin C induced HT29 cell apoptosis, which was blocked by concomitant catalase treatment [56].

Vitamin C also had a protective effect in a model of experimentally induced colon cancer in rats [57]. Daily administration of vitamin C to rats that received 1,2-dimethylhydrazine to induce colon cancer significantly reduced tumor markers (AFP, CEA, CA19-9) and protected the colon epithelium as observed histologically. In Gulo^−/−^ knockout mice, a mouse model where vitamin C synthesis is impossible, CMT-93 CRC tumor allograft growth was decreased by vitamin C supplementation, which also accelerated CMT-93 tumor regression [58]. Vitamin C also reduced the growth of Colon 26 tumor allografts [59]. In this model, vitamin C decreased intratumoral levels of VEGFA, VEGFD, HIF1α, and ROS.

Anti-proliferative and cytotoxic effects were further demonstrated in C2BBe1, WiDr and LS1034 colon cancer cells [60]. Vitamin C mechanisms of action included increased oxidative stress in C2BBe1 and WiDr cells as well as oxidative-stress-independent processes in LS1034 cells. Another study demonstrated the anti-cancer effects of vitamin C in WiDr colon cancer cells in culture or WiDr tumor xenografts [61]. NAC partially reversed these effects in vitro, suggesting that oxidative stress was at play. The pro-apoptotic effect of vitamin C was also found in HCT8 CRC cells [62]. Apoptosis was induced by release of calcium from the endoplasmic reticulum into the cytosol. A calcium chelator abolished the pro-apoptotic effect of vitamin C.

Besides vitamin C, the anti-cancer effect in CRC of its oxidized form DHA has also been investigated [63]. Treatment of LS174T CRC cells with DHA reduced cell proliferation and increased cell apoptosis. Of note, DHA also reduced the transcription of different stem cell markers, including *CXCR-4*, *Bmi-1*, *Sox-2*, and *Oct-4*, suggesting that CRC stem-like cells are also sensitive to DHA.

Studies examining vitamin C effects in vitro and in vivo are summarized in Table 1 and Table 2.

### 2.2. Factors Limiting the Anti-Cancer Effects of Vitamin C

The anti-cancer effects of vitamin C rely in part on the generation of extracellular H_2_O_2_, which will enter cancer cells and cause oxidative damage. Hence, molecules that interfere with this process might counteract the efficacy of vitamin C. In this context, iron plays an important role as it can decompose H_2_O_2_ via the Fenton reaction in the hydroxyl radical, which when present extracellularly is unable to produce cell damage. Since most cell media and sera used in cell culture contain lower levels of iron (0.25–5 µM) compared to plasma levels (10–30 µM), the influence of iron on the anti-cancer efficacy of vitamin C might have been overlooked in in vitro studies [68,69,70]. Within the scope of CRC, supplementation of culture medium with FeCl_3_ to a final concentration of 15 µM abolished the anti-cancer effect of vitamin C in DLD-1 and CT26 CRC cells. Conversely, addition of a polymeric iron chelator (PDFO) to culture medium recovered the anti-cancer efficacy of vitamin C in the presence of excess iron [64]. This effect was lost when catalase was added to the medium, highlighting the role of H_2_O_2_ in this process. Consistent with these in vitro observations, treatment of mice bearing CT26 tumor allografts with vitamin C and PDFO had higher anti-cancer effects compared to either treatment alone. More recently, these observations were confirmed, and culture medium supplementation with FeSO_4_ (10 µM) or FeCl_3_ (10 µM) prevented the cytotoxic effects of vitamin C in HCT116 and CT26 cells [65]. Similarly, ferric ammonium citrate or hemin reversed the anti-cancer effect of vitamin C in CT26 tumor allografts. Interestingly, whereas high levels of extracellular iron are detrimental to the anti-cancer effects of vitamin C, increasing intracellular iron has been shown to potentiate the effects of vitamin C in HCT116 and HT29 colon cancer cells [66]. Preexposure of HCT116 and HT29 with iron sucrose, which was removed prior to treatment with vitamin C, increased intracellular iron and the toxicity of vitamin C. In contrast, when HCT116 cancer cells were concomitantly exposed to iron sucrose and vitamin C, the effect of vitamin C was significantly reduced. Since CRC patients are often anemic and treated with iron sucrose, this study suggests that the treatment protocol should be carefully designed to increase intracellular but not extracellular tumor levels of iron.

Another potential limiting factor is the amount of vitamin C that can be delivered in cancer cells. This point was suggested by a study that showed that the extent of anti-cancer effects of vitamin C correlates with the expression level of SVCT-2 [67]. In this case, uptake of vitamin C was proportional to SVCT-2 expression. As a consequence, SW480, SW620, and LoVo colon cancer cells all expressing high amounts of SVCT-2 were sensitive to vitamin C, whereas HCT116, HCT15, and DLD-1 that express reduced levels of SVCT-2 were resistant. Even worse, vitamin C increased cell viability in CRC cells with low SVCT-2 expression, suggesting that vitamin C has a biphasic effect depending on its cellular uptake.

### 2.3. Combination of Vitamin C with Other Anti-Cancer Agents

Besides its anti-cancer efficacy as monotherapy, several studies have demonstrated the benefits of combining vitamin C with other treatment modalities in CRC. For example, in a mouse model of CRC induced by azoxymethane and dextran sodium, treatment with vitamin C in combination with irinotecan provides increased anti-cancer benefits compared to vitamin C or irinotecan alone [71]. The effect of vitamin C in combination with chemotherapies was further tested in three colon cancer cell lines, namely C2BBe1, LS1034, and WiDr. Vitamin C reduced IC_50_ of 5-fluorouracil, oxaliplatin, and irinotecan in every cell line [72]. Furthermore, in WiDr tumor xenografts, the anti-cancer effects of combining vitamin C with irinotecan or with oxaliplatin but not with 5-fluorouracil were significantly greater than those of either treatment alone. Finally, vitamin C increased the anti-cancer benefits of cisplatin in CT26 tumor cells in vitro [54].

In addition to increasing the anti-cancer benefits of chemotherapies, vitamin C enhances the effect of immunotherapy [73,74]. In fact, vitamin C delayed tumor growth of CT26 or MC38 CRC in immunocompetent but not in immunocompromised mice, showing that the anti-cancer properties of vitamin C require a functional immune system. Treatment of large CT26 colon cancer tumors (1000 mm^3^), with a volume known to be refractory to immune checkpoint therapy (ICT), with vitamin C in combination with the ICTs anti-CTLA-4 and anti-PD-1 led to tumor regression. Remission was observed in nearly 50% of treated animals that remained tumor free up to one year. More importantly, in a model of colon cancer harboring microsatellite instability, vitamin C in association with CTLA-4 or PD-1 blockade induced a full remission, while anti-CTLA-4 or anti-PD-1 used in monotherapy had no effect.

A fasting-mimicking diet had synergistic effects with vitamin C in *KRAS*-mutated CRC cells [75]. In vitro, starvation conditions (0.5 g/L glucose and 1% serum) or vitamin C had no effect or slightly increased cell death of *KRAS*-mutated HCT116, DLD-1 and CT26 colon cancer cells as compared to monotherapy. In contrast, when starvation was used in combination with vitamin C, nearly 100% cell death was achieved. Such combination therapy had no effects in *KRAS* wild-type HT29 and SW48 CRC cells. In vivo, a fasting-mimicking diet and vitamin C significantly reduced the growth of HCT116 tumor xenografts or CT26 tumor allografts. The anti-cancer efficacy was further significantly increased when a fasting-mimicking diet was used in combination with vitamin C. On the molecular level, starvation in combination with vitamin C increased intracellular ROS levels in HCT116 but not in SW48 or HT29 cells. NAC prevented vitamin C and starvation-mediated cell death, suggesting that ROS overproduction was responsible for cell death. Generation of hydroxyl radicals via the Fenton reaction, namely using ferrous iron and hydrogen peroxide, is a major contributor to oxidative damage. Consistent with that, ferrous iron levels were significantly increased in HCT116 cells following starvation and vitamin C cotreatment. Iron chelation with desferrioxamine rescued starvation and vitamin-C-mediated cell cytotoxicity. Additional analysis revealed that in *KRAS*-mutated colon cancer cells vitamin C upregulates the expression of ferritin in a HO-1-dependent mechanism, which reduces the labile iron pool. In contrast, in starvation conditions this mechanism is inhibited, thus increasing iron levels, leading to increased ROS generation. Finally, the anti-cancer effects of vitamin C and fasting were further increased in combination with oxaliplatin in HCT116 tumor xenografts and CT26 tumor allografts compared to monotherapies or bitherapies. This suggests that fasting and vitamin C represent a non-toxic strategy to increase the efficacy of chemotherapy in CRC patients.

Non-steroidal anti-inflammatory drugs exhibit chemopreventive activities towards CRC. In this context, combining vitamin C with sulindac showed increased cytotoxic effects compared to either treatment alone in HCT116 colon cancer cells. This effect occurred only in p53 wild-type HCT116 and was lost in p53-null HCT116 [76].

Vitamin C also provided benefit to anti-EGFR treatment such as cetuximab [77]. In fact, it increased the anti-cancer efficacy of cetuximab in cetuximab-sensitive colon cancer cells and rendered cetuximab-resistant colon cancer cells sensitive to cetuximab [44]. Indeed, whereas LoVo and SW480 cancer cells were resistant to cetuximab, combined treatment with vitamin C had synergistic effects. In addition, cetuximab reduced HT29 colon cancer cells by nearly 50% and they were further reduced when cells were treated in combination with vitamin C. These results suggest that, on one hand, vitamin C might sensitize resistant colon cancer cells to cetuximab, and on the other hand, it might increase the anti-cancer efficacy of cetuximab in cetuximab-sensitive colon cancer cells.

Additional observations further highlighted the benefits of a cetuximab–vitamin C combination treatment strategy in CRC expressing wild-type forms of *KRAS* and *BRAF* [78]. In particular, cotreatment of DiFi and CCK81 CRC cells with vitamin C and cetuximab prevented the emergence of acquired resistance over several months. In addition, combination therapy led to the disassembly of patient-derived *KRAS/BRAF* wild-type organoids and reduced the growth of patient-derived CRC tumor xenografts compared to vitamin C or cetuximab treatment alone. In this model, combined treatment with cetuximab–vitamin C also delayed emergence of resistance. Molecular analysis revealed that cetuximab impaired glycolysis and promoted ROS formation. Vitamin C further increased ROS production, which ultimately induced CRC cell death by ferroptosis.

EGFR targeting therapies provide no benefit in *KRAS/BRAF*-mutated CRC cells. Nevertheless, experimental evidence has demonstrated that adding vitamin C to these therapies overcomes resistance mediated by mutant *KRAS* in an SVCT2-dependent manner [79]. In this study, cetuximab did not alter cell viability of HCT8, HCT116, DLD-1, SW480 and SW620 CRC cells that express a mutant form of *KRAS*. However, combining vitamin C with cetuximab significantly increased cell death compared to vitamin C alone in DLD-1, SW480 and SW620 cells that all express SVCT-2. In contrast, this treatment combination had no effect in HCT8 and HCT116 cells in which SCVT-2 was not detected. The resistance of HCT8 and HCT118 to combined treatment could be overcome by transfecting an SCVT-2-expressing plasmid in HCT8 and HCT118. Similarly, siRNA-mediated downregulation of SCVT-2 expression in DLD-1, SW480 and SW620 cells induced resistance to vitamin C/cetuximab cotreatment. These observations were confirmed in vivo, where combining vitamin C reduced tumor xenograft growth of SW620 cells but not that of HCT116 cells. Hence, these data suggest that, in *KRAS* mutant CRC tumors, SCVT-2 might represent a marker for the treatment of vitamin C in combination with cetuximab.

Production of toxic ROS levels was also observed when vitamin C was associated with arsenic trioxide [80]. In this case, vitamin C and arsenic trioxide reduced the viability of SW620 and LoVo CRC cells in a synergistic manner. This effect was associated with ROS overproduction that led to apoptosis and pyroptosis.

CRC progression also arises from aberrant epigenetic mechanisms. In fact, DNA hypermethylation can lead to gene silencing of tumor suppressor genes, resulting in cancer progression. The group of ten-eleven translocation (TET) deoxygenases are important contributors to an active gene demethylation process that leads to transcriptional reactivation of silenced genes and therefore reduces tumor growth [81]. By using oxygen and ketoglutarate, TET enzymes are able to oxidize methylated cytosine, which at the end of a complex process will result in an unmodified cytosine. Importantly, vitamin C is necessary for an optimal TET activity. Mutation of the isocitrate dehydrogenase 1 enzyme, as found in some microsatellite-stable CRC, results in TET inactivation by accumulation of 2-hydroxyglutarate [82]. In this context, combining vitamin C with ML309, an inhibitor of mutant IDH enzymes, has been shown to rescue TET activity and consequently upregulate the expression of certain tumor suppressor genes in HCT116 cells that harbor a mutated allele of IDH1 [83]. In addition, cell viability was significantly reduced by the treatment combination compared to vitamin C or ML309 alone. Hence, this combination therapy might be of interest in IDH1/2-mutated CRC. Similarly, vitamin C was found to increase HCT116 cell apoptosis when used in combination with DNA-demethylating agents such as decitabine [84]. In this case, the combined treatment of vitamin C plus decitabine was associated with reexpression of the tumor suppressor protein p21.

Studies on benefits of vitamin C treatment in combination with other therapies are summarized in Table 3.

### 2.4. Lessons Learned from Preclinical Studies That Need to Be Addressed in Clinical Protocols

**Figure 3:**
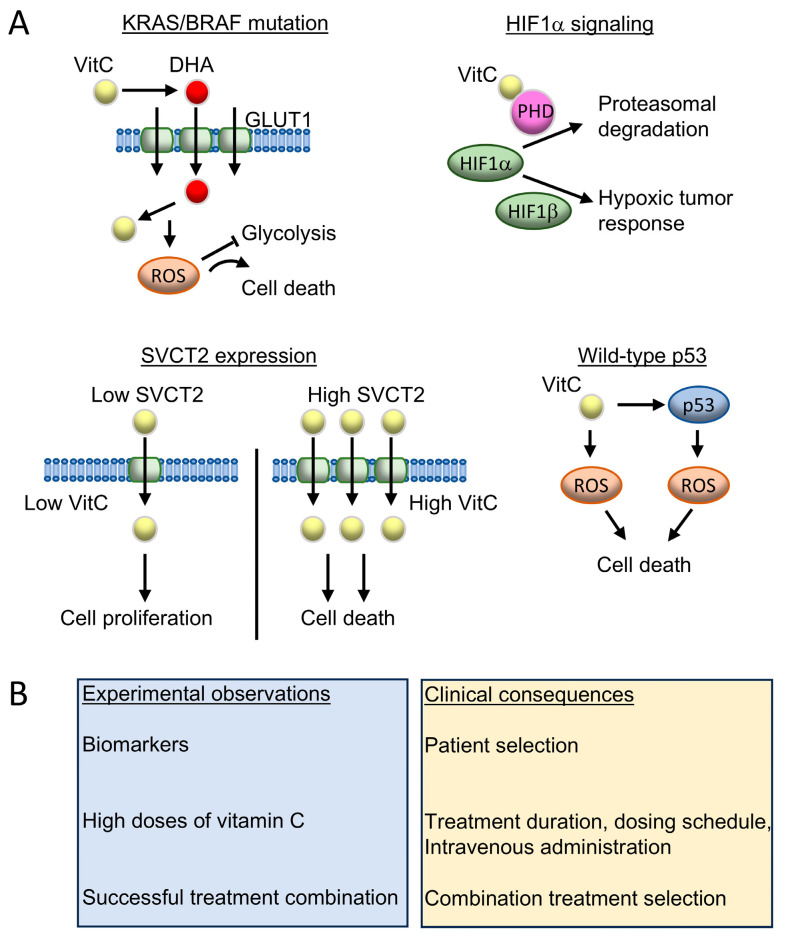
Preclinical observations and their clinical consequences. (**A**). Biomarkers of CRC sensitivity to vitamin C. CRC cells harboring *KRAS/BRAF* mutations express increased levels of GLUT1 and depend on glycolysis for their metabolic needs. Consequently, DHA uptake is increased, leading to higher ROS production that ultimately induce CRC cell death by blocking glycolysis. In hypoxic tumor microenvironment, HIF1α associates with HIF1β and induces the hypoxic tumor response leading to CRC progression. In normoxic conditions, prolylhydroxylases (PHDs), using vitamin C as a cofactor, hydroxylate HIF1α, resulting in its proteasomal degradation. Vitamin C uptake is increased in CRC cells that express high levels of SVCT2, which leads to cell death. In contrast, CRC cells that express low levels of SCVT2 display low levels of vitamin C that induce CRC cell proliferation. Vitamin C stimulates the pro-oxidative transcriptional program of wild-type p53, resulting in increased ROS production and CRC cell death. This effect is lost in presence of mutated p53. (**B**). The major experimental observations of an anti-cancer effect of vitamin C in CRC and their clinical consequences are enumerated.

Several key points from experimental studies need to be carefully addressed when translating experimental protocols into clinical practice (Figure 3).

Some tumors are more sensitive to vitamin C. For instance, tumors with *KRAS/BRAF* mutations rely highly on glycolysis for their metabolic needs, which is inhibited by oxidative stress generated by vitamin C. These tumor cells also have increased expression of GLUT1, resulting in increased uptake of DHA. The anti-cancer effect of vitamin C is also increased in CRC cells with intact p53 as it increases vitamin-C-mediated ROS formation. Since vitamin C is needed to induce HIF1α proteasomal degradation, CRC driven by HIF1α signaling is more sensitive to vitamin C treatment. Finally, CRC cells that express high levels of SCVT-2 benefit from the anti-cancer effects of vitamin C, as its uptake is increased. Therefore, CRC patient selection based on tumor biomarkers might be important.

Administration of high doses of vitamin C are necessary to achieve anti-cancer effects. Hence, determining dosages and treatment duration will be crucial in patients. In addition, anti-cancer effects of vitamin C are increased in combination with other therapies, including chemo-, immunotherapy or targeted therapies. Therefore, the ideal treatment combination needs to be identified for each CRC patient.

## 3. Anti-Cancer Efficacy of Vitamin C in CRC Patients

The effects of vitamin C on CRC mortality have been investigated in several clinical trials.

### 3.1. Initial Clinical Observations

Initial studies led to controversial results. Over fifty years ago, a case series with fifty advanced cancer patients among whom six had colon cancer and three had rectal cancer were treated in part with vitamin C, usually 10 g per day intravenously for ten days followed by oral administration of 10 g per day [85]. Two patients with colon cancer and one with rectal cancer did not respond to the treatment. The other patients exhibited benefits in terms of symptomatic relief and clinical improvement. One patient with colon cancer showed tumor regression. Since the study lacked any control patients, no conclusion on survival benefits could be made. Consequently, two other studies compared 100 patients with terminal cancer treated with vitamin C (50 patients were the ones described above) with 1000 similar untreated patients [86,87]. Among these 100 patients, 17 had colon cancer and seven had rectal cancer. All patients receiving ascorbic acid but one had longer survival times compared to control patients.

In contrast, a few years later, two randomized double-blind placebo-controlled studies failed to show any anti-cancer benefit of vitamin C. In the first study, patients with advanced cancers who were unsuitable for additional treatments either because of poor conditions or because of disease progression were randomized to either receive vitamin C orally or placebo. No survival benefit was observed between the groups [88]. Among the 123 patients who participated in the study, 50 had CRC. Although no subgroup analyses were performed according to the types of cancer, it did not appear that CRC patients survived longer following treatment with vitamin C. In the second study, only patients with advanced CRC who were not previously treated with chemotherapy were included [89]. This was chosen to exclude that prior health impairment induced by chemotherapy would diminish the benefits of vitamin C. In addition, CRC patients were selected, as at that time, no chemotherapy with survival benefit was available for these patients. Vitamin C treatment was given orally at 10 g daily in four separate doses. No survival benefit was observed between the groups. Whilst the different outcomes of these early studies could be caused by a lack of randomization, subsequent investigations showed that only intravenous administration of vitamin C generates high plasma concentrations that might produce anti-cancer benefits [90]. Indeed, in vitro evidence demonstrated that anti-cancer effects of vitamin C are mostly observed with concentrations higher than 1 mmol/L, which is not achieved following oral administration of vitamin C.

### 3.2. Case Report and Phase I–III Clinical Trials

Following the controversial initial observations, several clinical studies have further investigated the effects of vitamin C in CRC patients.

Consistent with an anti-cancer effect of vitamin C given intravenously, a case report described a 51-year-old man with advanced colon cancer who experienced a complete remission after surgery, chemotherapy consisting of 5-FU and leucovorin, and biweekly infusion of 100 g of vitamin C [91]. Of note, the patient had no toxic chemotherapy side effects while taking vitamin C. Interestingly, typical side effects of nausea, diarrhea, and stomatitis appeared whilst the patient was on vacation where chemotherapy was pursued but not vitamin C.

Following the observation that vitamin C has to be given intravenously to provide any benefits, phase I and II clinical trials were launched to assess the toxicity and maximal tolerable dose of vitamin C. A dose escalation study enrolled 24 patients (19/24 with CRC) with terminal cancers and no effective therapy available [92]. Patients had all received one or more chemotherapy regimens prior to the study. Vitamin C doses, given as continuous infusions, started at 150 milligrams per kilogram body weight per day and went up to 710 milligrams per kilogram body weight per day. Treatment was given over eight weeks unless progression of disease or adverse events occurred. Most side effects were minor with the most common being nausea, dry skin and mouth, edema, and fatigue. One subject developed a kidney stone after 13 days of treatment with a daily vitamin C dose of 290 milligrams per kilogram body weight. Of note, this patient had a prior history of kidney stones, hence other factors apart from vitamin C might have contributed to the kidney stone development. Another patient treated with 570 milligrams per kilogram body weight per day experienced hypokalemia, which might possibly have been caused by diarrhea rather than vitamin C. Most patients were vitamin C deficient prior to therapy with a mean plasma ascorbate level of 0.1 ± 0.2 mM compared to a mean plasma ascorbate concentration of 1.1 ± 0.9 mM during therapy (from 0.28 to 3.8 mM). Hence, a great variability of vitamin C pharmacokinetics exists and, therefore, vitamin C plasma concentrations should be closely monitored in cancer patients undergoing continuous vitamin C therapy. All but one patient with colon cancer had disease progression within eight weeks. One patient had stable disease. A reevaluation of this phase I study further revealed that continuous vitamin C therapy normalized several blood parameters, including neutrophil-to-lymphocyte ratio, lymphocyte count, and glucose levels [93]. The study further suggested that using high continuous vitamin C doses results in increased side effects without increasing plasma ascorbate levels.

A phase I–II study tested high-dose vitamin C in 14 cancer patients among whom three had advanced colon cancer and three had advanced rectal cancer [94]. In this protocol, patients received vitamin C three times a week, at least one day apart during chemotherapy weeks and every two days during weeks with no chemotherapy. Two of the CRC patients presented with low levels of vitamin C before entering the study. Administration of vitamin C showed no toxicity. One patient reported unpleasant but tolerable side effects. Four patients, two with colon cancer and two with rectal cancer, had disease progression despite vitamin C and chemotherapy treatments. Two patients, one with colon cancer and one with rectal cancer, experienced transient stable disease of 44 and 54 days.

A phase I trial tested safety and pharmacokinetics of high-dose intravenous vitamin C in advanced cancer patients refractory to any therapy [95]. Seventeen patients were enrolled, including four with colon cancer and one with rectal cancer. Five cohorts of three patients were determined with the first cohort receiving 30 g/m^2^ (1.5 g/kg = approximately 56 g/m^2^) of vitamin C administered at 1 g/min for four consecutive days/week for four weeks. The dose was augmented by 20 g/m^2^ for subsequent cohorts until reaching the maximal tolerated dose. Vitamin C was well tolerated. A C_max_ value of 49 mM was reached with 70 g/m^2^. Increasing vitamin C doses to 90 or 110 g/m^2^ did not increase C_max_. Stable concentrations at or above 10–20 mM were achieved over 5–6 h with 70–110 g/m^2^. No objective tumor response was observed.

Another phase I trial tested the safety of vitamin C in combination with mFOLFOX6 or FOLFIRI chemotherapies with or without bevacizumab in 30 patients with advanced CRC and six patients with advanced gastric cancer [96]. Findings were that vitamin C at 1.5 g per kilogram daily for three consecutive days in combination with chemotherapy every 14 days is well tolerated. The most common side effects attributed to vitamin C were headache and dry mouth. Toxicities of chemotherapies were reduced compared to those reported in other clinical trials with these chemotherapy regimens. For example, neutropenia was observed in 13.9% of patients compared to 30% in other studies. In addition, grade 3 neurotoxicity of oxaliplatin was found in 2.8% of patients instead of the expected 20% [97,98]. Of note, the maximal tolerable dose of vitamin C was not reached in this trial. Peak blood levels were 10–20 mmol/L of vitamin C, which are sufficient to inhibit cancer cell growth in vitro and tumor growth in vivo. The dose of 1.5 g/kg was however chosen for a phase III trial, as higher doses than 1.5 g/kg did not further increase the maximal plasma concentration of vitamin C nor the area under the curve. The median progression-free survival of all patients was 8.8 months. No difference was observed in metastatic CRC patients with wild-type *RAS/BRAF* compared to mutated *RAS* or *BRAF*.

Based on these results, a randomized phase III study investigated the anti-cancer efficacy of high-dose vitamin C in combination with chemotherapy (FOLFOX +/− bevacizumab) in metastatic CRC patients [99]. Vitamin C (1.5 g/kg) did not improve progression-free survival, the objective response rate and overall survival compared to controls. In addition, treatment-related adverse events were similar in the control and vitamin C groups. In other words, chemotherapy was not better tolerated in the presence of vitamin C. Nevertheless, subgroup analyses revealed that patients with *RAS* mutation had a significantly longer progression-free survival following vitamin C administration (9.2 versus 7.8 months; *p* = 0.01). In addition, in an unplanned subgroup, the progression-free survival of patients older than 55 years was also increased by vitamin C. Of note, in this study protocol, vitamin C was administered on three days of every cycle and was discontinued after six months, which might not be enough to induce the anti-cancer effects of vitamin C.

### 3.3. Ongoing Clinical Trials

Several clinical studies are currently testing the effect of vitamin C. A prospective single-arm trial evaluates high-dose vitamin C in combination with metformin in metastatic CRC with progression-free survival as the primary outcome [100]. Another randomized control study will compare the objective response rate, progression-free survival and overall survival of metastatic CRC patients expressing high levels of Glut3 treated with vitamin C in combination with adebrelimab and FOLFOX +/− bevacizumab versus treatment with FOLFOX +/− bevacizumab alone [101]. High expression of Glut3 is thought to increase the uptake of vitamin C and thus increase its efficacy. Vitamin C is further being tested preoperatively. A study is evaluating the safety and efficacy of high-dose vitamin C with mFOLFOX6 in locally advanced rectal cancer patients [102]. A phase Ib study has been designed to evaluate safety and effectiveness of botensilimab and balstilimab combined with a fasting-mimicking diet and high-dose vitamin C in patients with advanced CRC harboring *KRAS* mutations [103].

### 3.4. Other Studies Highlighting Benefits of Vitamin C in CRC Patients

A retrospective study found no statistically significant difference in terms of mortality and recurrence rates in CRC patients receiving vitamin C versus control CRC patients. In this study, 30 patients received vitamin C iv biweekly 1 g/kg for more than five weeks in addition to chemotherapy and surgery. They were compared to 150 CRC patients who had chemotherapy and surgery alone [104]. A major difficulty is to find the adequate vitamin C doses that provide concentrations with anti-cancer effects in tumors. In fact, the lack of anti-cancer efficacy of vitamin C might arise from inappropriate intratumoral levels of vitamin C. Most trials have measured blood concentrations of vitamin C, but whether these concentrations translate into anti-cancer vitamin C levels in tumors remains poorly investigated. This point is crucial, as a retrospective analysis of CRC samples found an association between tumor ascorbate content and increased disease-free survival in the first six years that followed surgery [105]. Interestingly, the survival advantage was correlated with the ascorbate amount in tumors but not in adjacent normal tissue. Also, tumor grade was negatively associated with the amount of tumor ascorbate, and tumors had reduced levels of ascorbate compared to normal tissue. In addition, there was an inverse correlation between HIF1α pathway activation and tumor size with tumor levels of ascorbate.

Since CRC frequently harbors poorly vascularized areas with abnormal vessels, intratumoral delivery of drugs might be particularly difficult. Nevertheless, high-dose infusion of vitamin C has been shown to increase tumor ascorbate levels in colon cancer patients [106]. Patients with colon cancer received infusion of vitamin C (nine patients; 25 g, four days before surgery, followed by 1 g/kg for three days) or no infusion (six patients). Tumor ascorbate levels increased from 15 ± 6 mg/100 g tissue in the biopsy sample to 28 ± 6 mg/100 g tissue in the surgical specimen. Significantly reduced expression of GLUT1 but not VEGF or CA-IX, all HIF-dependent proteins, was found in tumors of vitamin-C-treated patients compared to untreated patients. There was no significant reduction of the phosphorylated histone DNA repair protein gH2AX, a marker of oxidative-stress-mediated DNA damage. Hence, infusion of vitamin C increased intratumoral levels of vitamin C. Whether these levels are sufficient to generate anti-cancer benefits will be an important point to address.

Besides clinical trials, epidemiology studies have investigated the effect of vitamin C supplementation on CRC mortality. Interrogating 711,891 persons of the American Cancer Society’s Cancer Prevention Study-II cohort found no association between daily use of vitamin C and mortality of CRC [107]. However, subgroup analyses found that use of vitamin C supplements for ten or more years reduced risk of mortality of rectal cancer and decreased risk of CRC mortality before 65 years of age. A prospective observational study evaluated the effect of vitamin C supplementation on CRC-mediated mortality and non-CRC-mediated mortality [108]. Vitamin C supplementation was evaluated by a questionnaire with a yes or no answer with no further details regarding doses and administration routes. Analysis was performed for prediagnosis and postdiagnosis use of vitamin C. There were no statistically significant associations of pre- and postdiagnosis use of vitamin C with cancer- and non-cancer-related mortality. A similar study found that higher total vitamin C intake postdiagnosis was associated with lower CRC-related mortality in patients with stage I–III CRC harboring *KRAS* or *BRAF* mutations [109].

The use of high-dose vitamin C for CRC is not limited to clinical trials but is also widely used by complementary and alternative medicine practitioners [110]. Unfortunately, success or failure of these treatments is barely reported. Whilst clinical trials mostly concentrate on advanced forms of cancer, complementary and alternative medicine practitioners possibly also prescribe vitamin C to less advanced forms of cancer. It would be interesting to know if positive effects of vitamin C can be achieved in these cases.

Besides anti-cancer benefits, high-dose vitamin C might also ameliorate the quality of life of CRC patients. Consistent with this hypothesis, a study analyzed this point in 39 patients with terminal cancers who were not on chemotherapy and among whom nine had CRC. Patients received twice weekly ten grams of vitamin C intravenously and four grams orally daily for one week and quality of life was assessed by a questionnaire (EORTC QLQ-C30). Patients reported significantly lower scores for fatigue, pain, appetite loss, and nausea and had improvements in physical, social, emotional, and cognitive functions following vitamin C administration [111]. In addition, administration of high-dose vitamin C also reduces pain following colectomy in colon cancer patients. In a randomized double-blinded study, colon cancer patients who had colectomy reported less pain and consumed less morphine in the first 24 h postsurgery when receiving high-dose vitamin C compared to the ones who received placebo [112].

Finally, supplementation with vitamin C in metastatic CRC patients should be considered regardless of its anti-cancer effect. Low plasma concentrations of ascorbate were documented in CRC patients, suggesting an imbalance of antioxidants [92,113,114,115]. A study suggested low plasma vitamin C levels in colon but not rectal cancer [116]. Reduced plasma levels of vitamin C in CRC patients cannot be explained by reduced vitamin C intake, as intake was not statistically different between cancer patients and healthy controls [113,116], Reduced levels of vitamin C in CRC patients are more likely to result from an increased turnover due to increased oxidative stress in tumors [117]. Deficits of plasma vitamin C were not associated with increased mortality of CRC [113]. In contrast, the presence of CRC was an independent risk factor for reduced vitamin C levels. Since vitamin C plays a major role in human physiology, supplementation in these patients should be advocated.

In summary, several clinical studies have investigated the anti-cancer effects of vitamin C in CRC. No study except for one case report was able to demonstrate a long-lasting effect of vitamin C with a reduction of mortality of CRC, consistent with what was observed for other malignancies [118,119]. As of this date, only three randomized controlled trials have addressed the benefit of vitamin C in CRC. Two failed to find any anti-cancer benefits, since oral administration of vitamin C was used, which is not sufficient to achieve plasma high-dose vitamin C [88,89]. The third found a modest increase in progression-free survival of 1.4 months in patients with CRC harboring RAS mutation [99]. Several factors might explain this lack of effect. Most in vivo studies found a cytostatic but not a cytolytic effect of high-dose vitamin C as evidenced by a reduction of tumor growth rather than tumor regression in different models of CRC. Hence, the anti-tumor properties of vitamin C might be overestimated in preclinical analysis.

In addition, high-dose vitamin C is needed intratumorally to induce anti-tumor effects. In experimental studies, vitamin C was administered at least daily until the end of the study. In most clinical trials, vitamin C administration was not as frequent as in experimental settings, highlighting the difficulties to translate experimental protocols into clinical practice. A key point remains the determination of treatment duration and dosing schedule. Since intratumoral levels of vitamin C are not measured in patients, the question whether the treatment protocol generates anti-cancer concentrations of vitamin C will stay unanswered. Hence, a lack of anti-cancer effects of vitamin C either reflects a true inefficacy or a failure to obtain anti-cancer concentrations of vitamin C in tumors. Furthermore, the need to combine vitamin C with already existing treatments adds a layer of complexity in the elaboration of treatment protocols.

Preclinical experiments have demonstrated that tumors harboring certain types of mutations are particularly sensitive to vitamin C. This includes CRC harboring *KRAS/BRAF* mutations, CRC depending on HIF1α signaling, CRC expressing wild-type p53, and CRC expressing high levels of SCVT-2. Given this background, selecting patients based on tumor biomarkers will be of paramount importance in order to identify patients benefiting from vitamin C. Consistent with this, a phase III clinical study found a prolonged progression-free survival by vitamin C only in CRC with RAS mutations [99].

Experimental studies have demonstrated that the anti-cancer effect of vitamin C relies in part on increasing intracellular oxidative stress via the Fenton reaction, which can be influenced by iron [65,66]. These studies showed that high intracellular and low extracellular levels of iron enhance the anti-cancer effects of vitamin C. This point has not been addressed yet in clinical protocols and might be of importance, since CRC patients are often iron deficient.

Clinical studies showed that administration of high doses of vitamin C is safe with some minor adverse effects including nausea and vomiting. No increase in kidney calcium oxalate stone formation was reported, which could have been an issue since vitamin C is metabolized into oxalic acid. Administration of vitamin C remains, however, contraindicated in patients with glucose-6-phosphate dehydrogenase deficiency, since these patients are at risk of developing hemolysis.

Clinical trials investigating the effects of vitamin C on CRC are summarized in Table 4.

## 4. Conclusions

CRC remains a major cause of cancer-related death, highlighting the need to explore additional treatment strategies. In this context, several experimental studies have demonstrated the anti-cancer potential of high-dose vitamin C in CRC. In particular, CRC harboring *KRAS* or *BRAF* mutations has been shown to be vulnerable to vitamin C treatment. Mechanistic analysis revealed that vitamin C increases oxidative stress, which is coupled with altered glycolysis and decreased ATP production, leading ultimately to cell death. Since the high metabolic needs of *KRAS*- or *BRAF*-mutated cancer rely mostly on glycolysis, these mechanistic insights may explain why high-dose vitamin C provides strong benefits in these tumors. Importantly, some studies have suggested that the effects of vitamin C might be dampened by certain factors. Among these, iron plays a crucial role for the generation of oxidative stress by vitamin C, and finding the right extracellular/intracellular levels of iron needed will be an important question to address.

In addition to being used in monotherapy, vitamin C provides additional anti-cancer benefits to currently existing CRC treatments. In particular, vitamin C increases the efficacy of chemotherapies such as oxaliplatin and irinotecan, of immunotherapies, including checkpoint inhibitors anti-CTLA-4 and anti-PD-1, and of EGFR inhibitor cetuximab. Additional studies should further elucidate whether anti-angiogenic drugs, which are widely used in CRC treatments, might also benefit from vitamin C cotreatment.

Besides experimental evidence, the anti-cancer efficacy of vitamin C towards CRC has also been investigated in patients. Overall, most studies failed to show any relevant anti-cancer benefits of vitamin C in CRC. Clinical trials have demonstrated that high-dose vitamin C is well tolerated. However, anti-cancer benefits of vitamin C in CRC have been limited to case reports. To date, phase III clinical trials have failed to demonstrate anti-cancer efficacy of vitamin C with the exception of patients with *KRAS* mutations who demonstrated a longer progression-free survival with vitamin C treatment. Ongoing and future trials will further help identify patients and protocols where vitamin C provides anti-cancer effects. Key investigations will be to identify vitamin C doses that generate anti-cancer levels of vitamin C in tumors. Importantly, a big variability of vitamin C pharmacokinetics exists between patients so plasma levels of vitamin C following treatment should be closely monitored. In addition, most studies have focused on plasma levels, which might not reflect intratumoral concentrations. Investigating intratumoral concentrations of vitamin C might be difficult as cancer samples under therapy would be required, exposing the patient to possible additional tumor biopsies. Finally, it might be judicious to test vitamin C in less advanced forms of CRC, since vitamin C is well tolerated and benefits might be present in non-advanced cancers.

## Figures and Tables

**Table 1:** Anti-cancer effects of vitamin C in CRC cell lines. Cell lines, as specified in the first column, are highlighted in bold and followed by the different genetic alterations of these cell lines.

Cell Type	Treatment	Main Analysis	Results	References
**HCT116, DLD1** WT *KRAS* or Mut *KRAS* G13D **VACO432, RKO** WT *BRAF* or Mut *BRAF* V600E	Vitamin C 0.125–2 mM 24–48 h	Vitamin C uptake analysis Cell viability assay Liquid chromatography tandem mass spectrometry metabolite analysis ROS measurement	CRC cells preferentially uptake DHA via Glut1. Import is faster in mutated CRC cells. Vitamin C decreases mutant cell growth and colony formation and increases mutant cell apoptosis compared to wild-type counterparts. Vitamin C increases intracellular oxidative stress resulting in reduced glycolysis and energy crisis.	[40]
**HT29** *TP53* R273H *BRAF* V600E	Vitamin C 1–5–10 mM for 2 h. Measure of cancer cell viability 46 h later	EC50 determination Cell viability assay Capillary electrophoresis time-of-flight mass spectrometry metabolite analysis	HT29 EC50 > 10 mM. NAC and GSH reduce the cytotoxic effects of vitamin C. GSH/GSSG ratio is decreased, confirming increased cellular oxidative stress induced by vitamin C. Energy flux in glycolysis and TCA cycle is disrupted by vitamin C, resulting in reduced ATP production.	[41]
**HT29** *TP53* R273H *BRAF* V600E	Vitamin C 3–6 mM for 4–24 h	Cell viability assay Liquid chromatography tandem mass spectrometry metabolite quantification	Vitamin C reduces HT29 viability. Vitamin C disrupts glycolysis and increases the pentose phosphate pathway.	[42]
**HCT116** *KRAS* G13D *PI3KCA* H1047R **FHC** (colon epithelial cells)	Vitamin C 0.625–20 mM for 1 h. Proliferation measured 24 h later	Cell viability assay Cell proliferation assay Lactate, ATP glucose measurements	Vitamin C reduces HCT116 cell viability and proliferation. Vitamin C has no or little effect on FHC cell proliferation. Vitamin C reduces glucose consumption, lactate and ATP production in HCT116 cells but not in FHC colon epithelial cells.	[43]
**SW480** *KRAS* G12V *TP53* R273H;P309S **HT29** *TP53* R273H *BRAF* V600E **LoVo** *KRAS* G13D; V14A **HCEC** immortalized human colonocytes	2–5–10 mM for 2–36 h	EC50 determination Cell viability assay Apoptosis measurement ROS measurement	EC50 < 10 mM for SW480 and LoVo cancer cells. No effect of vitamin C on HCEC at 10 mM. Vitamin C reduces cancer cell viability but had no effects on colonocytes. Vitamin C inhibits Ras/Mek/Erk signaling pathways leading to reduced Glut1 and PKM2 expression and impaired glycolysis. Vitamin C reduces ROS levels, which leads to Ras inhibition via detachment from cell membrane.	[44]
**SW480** *KRAS* G12V *TP53* R273H;P309S **DLD 1 cell line** *KRAS* G13D *TP53* S241F *PI3KCA* E545K; D549N **HCEC** immortalized human colonocytes	Vitamin C 1–10 mM for 4 to 24 h	Cell viability assay Apoptosis assay HIF1α stability and transcriptional activity	Vitamin C reduces cancer cell viability but had no effect on colonocytes. Vitamin C reduces, in hypoxic conditions, HIF1α stability and transcriptional activity, leading to reduced Glut1 and PDK1 expression and ATP production.	[45]
**WiDr** *TP53* R273H *BRAF* A600E; T119S	Vitamin C 0.05–0.25–1 mM overnight	HIF1α stability and transcriptional activity	Vitamin C inhibits HIF1α expression induced by CoCl_2_, DFO, or 5% O_2_.	[47]
Lentivirus-mediated active HIF1α or HIF2α subunit overexpression **SW480** *KRAS* G12V *TP53* R273H; P309S **HCT116** *KRAS* G13D *PI3KCA* H1047R	Vitamin C 8 mM for HCT116 cells and 16 mM for SW480 cells for 1 h	Cell viability assay	Overexpression of a constitutively active HIF1α or HIF2α subunit in HCT116 or SW480 colon cancer cells increases cell death induced by vitamin C. DMOG-mediated HIF signaling pathway activation increases HCT116 and SW480 cells’ susceptibility to vitamin C cytotoxicity.	[48]
**HCT116** *KRAS* G13D *PI3KCA* H1047R	Vitamin C treatment for 24 h (0.5–10 mM)	MTT cell viability assay	Vitamin C reduces viability of HCT116 cells that express wild-type p53 compared to HCT116 that do not express p53. Vitamin-C-mediated H_2_O_2_ production is increased in p53-expressing HCT116 cells. Vitamin C favors a pro-oxidant transcriptional network in p53-positive but not in p53-negative HCT116 cells.	[49]
**HT29** *TP53* R273H *BRAF* V600E **CT26** *KRAS* G12V	Vitamin C 0–20 mM for 2 h, cell viability determined after 24–48 h	EC50 determination Clonogenic assay	IC50 < 5 mM for CT26. IC50 > 20 mM for HT29. Colony growth of the human colon cancer cell line HT29 was not affected by ascorbic acid.	[50,51]
**SW480** *KRAS* G12V *TP53* R273H; P309S **HT29** *TP53* R273H *BRAF* V600E **LS174T** *KRAS* G12D *BRAF* D211G *PI3KCA* H1047R	Vitamin C 0.1–20 mM for 24–72 h	Cell cycle analysis Cell proliferation	Vitamin C reduces CRC cell proliferation and increases CRC cell apoptosis. Vitamin C downregulates long non-coding RNA MALAT. CRC cells that express high levels of MALAT1 are more sensitive to vitamin C.	[52]
**CT26** *KRAS* G12V	Vitamin C 200–500–1000 µg/mL for 24–72 h	MTT assay Annexin V/PE flow cytometry analysis	Vitamin C reduces CT26 proliferation and increases CT26 apoptosis. Vitamin C effects partially reversed by NAC.	[54]
**HT29** *TP53* R273H *BRAF* V600E	Vitamin C 3 mM for 24 h	Cell count Light microscope	Vitamin C reduces HT29 cell growth and viability.	[55]
**HT29** *TP53* R273H *BRAF* V600E	Vitamin C 5–10–15–20 mM 4 h–24 h–32 h	MTT assay Cell count Electronic microscope Cell cycle analysis Clonogenic assay	Vitamin C induces HT29 cell apoptosis. The effect is abolished by catalase.	[56]
**C2BBe1** **WiDr** *TP53* R273H *BRAF* A600E; T119S **LS1034** *TP53* G245S *KRAS* A146T	Vitamin C 0.25–20 mM for 24–96 h	Cell proliferation assay Clonogenic assay Apoptosis assay	Vitamin C reduces proliferation and survival of CRC cells. Vitamin C mechanisms of action included increased oxidative stress in C2BBe1 and WiDr cells as well as oxidative-stress-independent processes in LS1034 cells.	[60]
**WiDr** *TP53* R273H *BRAF* A600E; T119S	Vitamin C 0.5–45 mM for 1–4 h; analysis 24–96 h later	MTT assay Colonogenic assay Apoptosis assay	Vitamin C decreases WiDr proliferation and survival. Vitamin C increases oxidative stress.	[61]
**HCT8**	Vitamin C 2 and 4 mM for 24 h	Apoptosis assay	Vitamin C increases HCT8 apoptosis. The effect is blocked by calcium chelator.	[62]
**LS174T** *KRAS* G12D *BRAF* D211G *PI3KCA* H1047R	DHA 100–500–1000 µM for 48 h	Cell count Proliferation assay Apoptosis assay	DHA reduces LS174T cell proliferation and induces LS174T cell apoptosis. DHA reduces transcription of pluripotency genes *CXCR4*, *Bim-1*, *Sox-2* and *Oct-4*.	[63]
**DLD 1 cell line** *KRAS* G13D *TP53* S241F *PI3KCA* E545K; D549N **CT26** *KRAS* G12V	Vitamin C 0.1–10 mM for 2 h	Cell viability assay Apoptosis assay	Vitamin C reduces CRC cells’ viability. The effect is lost when catalase is added to the medium, highlighting the role of H_2_O_2_ in this process. Vitamin C anti-cancer effect is abolished by adding FeCl_3_ to culture medium. Conversely, addition of polymeric iron chelators to culture medium recovered the anti-cancer efficacy of vitamin C in presence of excess iron.	[64]
**HCT116** *KRAS* G13D *PI3KCA* H1047R **CT26** *KRAS* G12V	Vitamin C 2 mM for 4 h		Vitamin C reduces CRC cells’ viability. Vitamin C effects are abolished by supplementing the culture medium with FeSO_4_ or FeCl_3_.	[65]
**HCT116** *KRAS* G13D *PI3KCA* H1047R **HT29** *TP53* R273H *BRAF* V600E	Vitamin C 10 mM for 2 h	Clonogenic assay	Vitamin C reduces CRC cell survival. The effect is blocked by catalase added in the culture medium or overexpressed intracellularly. Whereas high levels of extracellular iron are detrimental to the anti-cancer effects of vitamin C, increasing intracellular iron potentiates the effects of vitamin C. Preexposure of HCT116 and HT29 with iron sucrose, which is removed prior to treatment with vitamin C, increased intracellular iron and the toxicity of vitamin C.	[66]
**SW480** *KRAS* G12V *TP53* R273H;P309S **SW620** *KRAS* G12V *TP53* R273H;P309S **HCT15** *KRAS* G13D *TP53* S241F *PI3KCA* E545K; D549N **HCT116** *KRAS* G13D *PI3KCA* H1047R **DLD1** *KRAS* G13D *TP53* S241F *PI3KCA* E545K; D549N **LoVo** *KRAS* G13D; V14A **CoLo205** *BRAF* V600E *TP53 Y103fs*	Vitamin C 10 µM, 1 mM for 4 h. Cell viability measured 20 h later	Cell viability assay	Intracellular uptake of vitamin C is proportional to SVCT-2 expression. Vitamin C reduces viability of high-SVCT-2-expressing CRC cells (SW480, SW620 and LoVo). Low-dose vitamin C (<0.1 mM) increases viability of low-SCVT-2-expressing CRC cells (HCT116, HCT15 and DLD1), suggesting that vitamin C has a biphasic effect depending on its cellular uptake.	[67]

**Table 2:** Anti-cancer effects of vitamin C in vivo.

Mouse Model	Treatment	Main Analysis	Results	References
HCT116 and VACO432 tumor xenografts	Vitamin C 4 g/kg ip twice a day for 3–4 weeks	Tumor growth	Vitamin C reduces tumor xenograft growth. NAC abolishes the effect of vitamin C.	[40]
Transgenic, tamoxifen-induced mouse model of intestinal cancer (*Apc^flox/flox^/Kras^G12D^* versus *Apc^flox/flox^*)	Vitamin C 4 g/kg ip daily for 5–6 weeks	Polyp number and size	Vitamin C reduces the number and size of intestinal polyps in *Apc ^flox/flox^/Kras^G12D^* mice compared to *Apc^flox/flox^* mice. Tumors from *Apc ^flox/flox^/Kras^G12D^* mice had higher Glut1 expression and increased Vitamin C uptake compared to tumors from *Apc^flox/flox^* mice.	[40]
HCT116 tumor xenografts	Vitamin C 4 g/kg ip or orally twice a day for 21 days	Tumor growth Ki67 histology Measurement of TNFα, IL-6 and IL-10 levels	Vitamin C reduces tumor growth and tumor cell proliferation, increases necrosis and fibrous connective tissue. Vitamin C reduces serum and intratumoral TNFα, IL-6 levels and increases serum and intratumoral IL-10 levels.	[43]
SW480 tumor xenografts	Vitamin C 4 g/kg ip daily for 15 days	Tumor growth Glut1, PKM-2 and phospho-Erk histology	Vitamin C reduces tumor growth. Vitamin C reduces Glut1, PKM-2 and phospho-Erk tumor expression.	[44]
SW480 tumor xenografts	Vitamin C 4 g/kg ip daily for 15 days	Glut1 and PDK-1 histology	Vitamin C reduces Glut1 and PDK-1 tumor expression.	[45]
p53^+/+^ HCT116 tumor xenograft and p53^−/−^ HCT116 tumor xenograft	Vitamin C 4 g/kg ip daily for 15 days	Tumor growth	Vitamin C reduces p53^+/+^ HCT116 tumor xenograft growth and has no effect on p53^−/−^ HCT116 tumor xenograft.	[49]
LS174T tumor xenograft Intraperitoneal injection of LT174T cells	High-dose vitamin C 4 g/kg ip every two days Low dose 100 mg/kg	Tumor growth CD31 histology LncRNA MALAT1 expression levels	High-dose vitamin C decreases tumor growth, tumor angiogenesis and metastasis formation. Vitamin C decreases MALAT1 expression.	[52]
CT26 tumor allograft	Three consecutive days of increasing doses of vitamin C Low-dose protocol: 1–2–3 mg/kg High-dose protocol: 2–4–6 mg/kg Intratumoral delivery	Tumor growth	Vitamin C decreases CT26 tumor allograft growth.	[54]
CT26 tumor allograft	Vitamin C 1.5 mg/g ip every three days for 30 days	Tumor weight Proteomic analysis	Vitamin C reduces tumor weight in one set of experiments but had no significant effect in a second set.	[53]
HT29 tumor xenograft	Vitamin C ip daily Low dose 100 mg/kg or 15 mg/kg High dose 1 g/kg	Tumor growth and weight mRNA for tRNA synthetase and translation initiation factor subunit expression	High-dose vitamin C reduces tumor growth and tumor weight. High-dose vitamin C decreases expression of RNA coding for tRNA synthetase and translation initiation factor subunits.	[55]
Chemical-induced colon cancer in rats	Vitamin C 200 mg/kg orally daily for 6 weeks	Tumor histology Serum concentrations of tumor markers (AFP, CEA, CA19-9)	Vitamin C reduces colon epithelium destruction and leucocyte infiltration. Vitamin C reduces serum concentrations of tumor markers.	[57]
CMT-93 tumor allograft in Gulo^−/−^ mice	Vitamin C supplementation in drinking water (3300 mg/L, 330 mg/L, 33 mg/L)	Tumor growth	Following initial growth, CMT-93 tumors regress within two weeks. High-dose vitamin C (3300 mg/L) reduces tumor growth and accelerates tumor regression compared to low-dose vitamin C (330 mg/L and 33 mg/L).	[58]
Colon 26 tumor allograft	Vitamin C 4 g/kg orally daily for 14 days	Tumor growth Western blot analysis of p53, VEGFA, VEGFD and HIF1α ROS measurement	Vitamin C reduces the growth of Colon 26 tumor allograft. Vitamin C reduces intratumoral levels of ROS, HIF1α and VEGF and decreases levels of endostatin and p53.	[59]
WiDr tumor xenografts	Vitamin C 150 mg/kg ip daily for 12 days	Tumor growth	Vitamin C reduces WiDr tumor xenograft growth.	[61]
DLD-1 tumor xenografts CT26 tumor allografts	Vitamin C 10 mg iv daily for 12 days	Tumor growth TUNEL assay	Vitamin C has no effect on the growth of DLD-1 tumor xenograft or CT26 tumor allograft. Vitamin C in combination with the iron chelator PDFO reduces the growth of DLD-1 tumor xenograft or CT26 tumor allograft. Vitamin C in combination with PDFO increases tumor cell apoptosis in DLD-1 tumor xenografts.	[64]
CT26 tumor allograft	Vitamin C 4 g/kg ip daily for 14 days	Tumor growth MDA measurement	Vitamin C reduces the growth of CT26 tumor allograft and MDA tumor levels. The effects of vitamin C are abolished by the iron donors ferric ammonium citrate and hemin.	[65]

**Table 3:** Anti-cancer effects of vitamin C in combination treatment.

Cell Type/ Mouse Model	Treatment	Main Analysis	Results	References
Mouse model of chronic inflammation-associated CRC induced by azoxymethane and dextran sodium	Vitamin C: 100 mg/day 5 days a week orally for 20 weeks Irinotecan: 60 mg/kg/day ip for four cycles of three weeks’ treatment followed by two weeks off treatment	Tumor number and area ROS and IL-6 plasma levels Colon caspase-1 expression Colon neutrophil and TUNEL staining	Vitamin C in combination with irinotecan provides increased anti-cancer benefits compared to vitamin C or irinotecan alone. Combined treatment increases neutrophil infiltration and caspase-1 expression in colon.	[71]
C2BBe1, LS1034, WiDr	Vitamin C 0.15–17 mM 5-Fluorouracil 0.5–480 mM Oxaliplatin 1–120 mM Irinotecan 5–200 mM for 24–96 h	IC_50_ determination Proliferation assay Cell cycle analysis	Vitamin C reduces IC_50_ of 5-fluorouracil, oxaliplatin and irinotecan in every cell line.	[72]
WiDr tumor xenograft	Vitamin C 150 mg/kg for 14 days 5-Fluorouracil 2 mg/kg Oxaliplatin 4 mg/kg Irinotecan 100 mg/kg	Tumor growth Immunohistochemistry of KI-67 expression	Vitamin C increases the anti-cancer efficacy of oxaliplatin or irinotecan but not 5-fluorouracil.	[72]
CT26	Vitamin C 200 mg/mL Cisplatin 1 mg/mL for 48 h	Apoptosis assay	Vitamin C increases the pro-apoptotic effect of cisplatin.	[54]
CT26 and MC38 tumor allografts	Vitamin C 4 g/kg per day for 5 days per week ip Anti-CTLA-4 200 mg ip Anti-PD-1 250 mg ip for 4 days	Tumor growth Mouse survival	Vitamin C delays tumor growth of CT26 or MC38 CRC in immunocompetent but not in immunocompromised mice. Treatment of large CT26 colon cancer tumors with vitamin C in combination with anti-CTLA-4 and anti-PD-1 treatments led to tumor regression.	[73]
HCT116, DLD-1, CT26, SW48 and HT29	Vitamin C 350–700 mM for 48 h Starvation condition: 0.5 g/L glucose and 1% serum	Cell viability ROS measurement Intracellular ferrous iron measurement	Fasting-mimicking diet had synergistic effect with vitamin C in *KRAS*-mutated CRC cells. NAC prevents this effect. Vitamin C treatment increases ferritin expression, which reduces ferrous iron pool and thus limits vitamin-C-mediated ROS production. Fasting-mimicking diet prevents vitamin-C-mediated ferritin upregulation and thus augments vitamin C anti-cancer effects.	[75]
HCT116 tumor xenograft CT26 tumor xenograft	Vitamin C treatment 4 g/kg twice a day ip for the last day of the first fasting-mimicking diet cycle to the end of the experiment Fasting-mimicking diet for two cycles of three days per week Oxaliplatin 10 mg/kg ip once every 15 or 11 days	Tumor growth Mouse survival	Fasting-mimicking diet potentiates the anti-cancer effects of vitamin C. The anti-cancer efficacy of vitamin C in combination with fasting-mimicking diet is further potentiated by oxaliplatin.	[75]
HCT116	Vitamin C 0.1–0.5 mM for 48 h Sulindac 0.1–0.5 mM	Cell viability Apoptosis assay ROS measurement	Vitamin C with sulindac shows increased cytotoxic effects compared to either treatment alone in p53 wild-type HCT116 cells but not in p53-null HCT116. Vitamin C and sulindac increase ROS levels. The cytotoxic effect of the combined treatment is prevented by NAC.	[76]
HT29 SW480 LoVo	Vitamin C 5 mM for 12 h Cetuximab 0.4 mM	Cell count	Vitamin C increases the anti-cancer effects of cetuximab in cetuximab-sensitive CRC cells. Vitamin C sensitizes cetuximab-resistant CRC cells to cetuximab.	[44]
CCK-81 DiFi	Vitamin C 1 mM for two weeks Cetuximab 50–100 mg/mL	Cell viability Clonogenic assay SILAC analysis Seahorse metabolic analysis	Combined treatment vitamin C–cetuximab reduces CRC cell growth and delays the emergence of resistant cells. Combined treatment induces the disassembly of patient-derived *RAS*/*BRAF* wild-type CRC organoids. Whereas cetuximab disrupts glycolysis and induces modest increase in ROS, addition of vitamin C impairs iron metabolism and further increases ROS, which leads to ferroptosis.	[78]
*RAS/BRAF* wild-type CRC patient-derived tumor xenografts	Vitamin C 4 g/kg ip daily five days a week for up to twelve weeks Cetuximab 10 mg/kg ip twice a week	Tumor growth	Vitamin C in combination with cetuximab reduced the growth of patient-derived tumor xenografts compared to vitamin C or cetuximab treatment alone. Combined treatment delays the formation of resistance.	[78]
HCT8, HCT116, DLD-1, SW480, SW620	Vitamin C 0.3–0.7 mM Cetuximab 0.5 mM	Cell death analysis Colony formation assay ROS level determination	Combining vitamin C with cetuximab significantly increases cell death compared to vitamin C or cetuximab alone in DLD-1, SW480 and SW620 cells that all express SVCT-2. No effect in HCT8 and HCT116 cells in which SCVT-2 was not detected. Resistance of HCT8 and HCT116 cells to combined treatment could be overcome by transfecting an SCVT-2-expressing plasmid in HCT8 and HCT116.	[79]
SW620 and HCT116 tumor xenografts	Vitamin C 0.5 g/kg ip daily for 15 days Cetuximab 50 mg/kg ip three times a week	Tumor growth	Combining vitamin C with cetuximab reduces tumor growth compared to either treatment alone in tumor xenografts that express SCVT-2. Combined treatment has no significant effect in tumor xenografts that do not express SCVT-2.	[79]
SW620, LoVo	Vitamin C 2 mM for 24 h Arsenic trioxide 2 mM	Cell viability assay Colony formation assay ROS level determination	Cotreatment vitamin C and arsenic trioxide increases CRC cell death compared to either treatment alone. The anti-cancer effect of cotreatment is associated with ROS overproduction that leads to apoptosis and pyroptosis.	[80]
HCT116	Vitamin C 1 mM for 24 h ML309 5–50 mM	Cell viability assay Apoptosis assay	Combining vitamin C with ML309 increases HCT116 cell apoptosis compared to either treatment alone in HCT116 cells that harbor *IDH1* allele mutation.	[83]
HCT116	Vitamin C 50 mM for 72 h Decitabine 1 mM	Cell viability assay Apoptosis assay	Vitamin C in combination with decitabine increases HCT116 cell apoptosis compared to vitamin C or decitabine alone. Treatment with vitamin C and decitabine is associated with reexpression of the tumor suppressor protein p21.	[84]

**Table 4:** Anti-cancer effects of vitamin C in clinical trials.

Study Type	Patients	Treatment of Vitamin C	Effects/Results	References
Case study	Total advanced cancer patients: 50 Colon cancer: 6 Rectal cancer: 3	10 g/day iv for 10 days and orally thereafter	Three patients (two colon cancer, one rectal cancer) showed no response. The other CRC patients exhibited clinical improvements. One patient with CRC had tumor regression.	[85]
Case–control	Total advanced cancer patients treated with vitamin C: 100 (50 are those described in [85]) Colon cancer: 13 Rectal cancer: 7 Matched untreated control cancer patients: 1000	10 g/day iv for 10 days and orally thereafter	Average days of survival 7.61 times and 4.1 times higher respectively in treated colon cancer patients and rectal cancer patients compared to matched control patients. 12/13 treated colon cancer patients lived longer than matched controls. 5/7 treated rectal cancer patients lived longer than matched controls.	[86]
Case–control	Total advanced cancer patients treated with vitamin C: 100 (most are the same as in [86]) Colon cancer: 17 Rectal cancer: 7 Partly new set of matched untreated control cancer patients compared to [86]: 1000	10 g/day iv for 10 days and orally thereafter	Mean survival times were significantly longer in colon cancer patients and rectal cancer patients compared to matched untreated patients. Quality of life of the patients is improved by the administration of vitamin C.	[87]
Randomized controlled double-blind study	Total advanced cancer patients treated with vitamin C: 60 CRC: 24 Total advanced cancer patients treated with placebo: 63 CRC: 26	10 g/day in 4 doses orally	No therapeutic benefit of vitamin C treatment. No benefits of vitamin C treatment in symptoms, performance status, appetite or weight.	[88]
Randomized controlled double-blind study	Total: 100 patients with advanced CRC 49 receiving placebo 51 receiving vitamin C	10 g/day in 4 doses orally	Vitamin C therapy showed no advantage with regard to either the interval between the beginning of treatment and disease progression or patient survival. Vitamin C therapy is not effective against advanced CRC regardless of any prior chemotherapy.	[89]
Case report	1 patient with advanced colon cancer	100 g twice a week iv	Complete remission after surgery, chemotherapy and vitamin C. No toxic chemotherapy side effects whilst under vitamin C substitution.	[91]
Phase I and II	Total: 24 advanced cancer patients 17 advanced colon cancer 1 advanced rectal cancer	Continuous iv ascorbic acid, 150 mg per kilogram body weight per day and went up to 710 mg per kilogram body weight per day (10–50 g) administered by injection pumps for up to 8 weeks	Minor side effects. Mean plasma ascorbate concentration was 1.1 ± 0.9 mM (from 0.28 to 3.8 mM) per patient. One patient with colon cancer had stable disease. The other 17 patients with CRC had tumor progression.	[92]
Phase I and II Reevaluation of [92]	17 patients with advanced colon cancer	Continuous iv ascorbic acid, 150 mg per kilogram body weight per day and went up to 710 mg per kilogram body weight per day (10–50 g) administered by injection pumps for up to 8 weeks	Normalization of several blood parameters by vitamin C including lymphocyte count, glucose levels and neutrophil-to-lymphocyte ratio.	[93]
Phase I and II	Total: 14 advanced cancer patients 3 advanced colon cancer 3 advanced rectal cancer	1.5 g iv per kilogram body weight for BMI < 30 kg/m^2^ and per corrected body weight, corresponding to a BMI of 24 kg/m^2^ for patients with BMI > 30 kg/m^2^ over a period of 90 to 120 min 3× per week	Minor side effects. Four patients (two colon, two rectum) showed disease progression under vitamin C and chemotherapy. Two patients (one colon, one rectum) had stable disease for 44 and 54 days.	[94]
Phase I	Total: 17 advanced cancer patients 4 advanced colon cancer 1 advanced rectal cancer	Dose escalation starting at 30 g/m^2^ and increased by 20 g/m^2^ for subsequent groups until MTD Administration iv at 1 g/min for 4 consecutive days per week for 4 weeks	No major side effects. Cmax vitamin C: 49 mM achieved with 70 g/m^2^. No tumor response.	[95]
Phase I	Total: 36 advanced cancer patients 30 advanced CRC	Vitamin C in combination with mFOLFOX6 or FOLFIRI chemotherapies with or without bevacizumab Dose-escalation phase: patients received vitamin C (0.2–1.5 g/kg, 3-h infusion, once daily, days 1–3) with mFOLFOX6 or FOLFIRI in a 14-day cycle until the maximum tolerated dose (MTD) was reached	MTD of vitamin C was not reached in this trial. Cmax vitamin C: 10–20 mM. Toxicities of chemotherapies were reduced compared to those reported in other clinical trials with these chemotherapy regimens. No difference in treatment efficacy between *RAS*/*BRAF* wild-type and *RAS*- or *BRAF*-mutated tumors.	[96]
Phase III clinical trial, randomized, open-label, multicenter, (VITALITY study)	Total: 442 advanced CRC patients Control (221 patients): FOLFOX+/−bevacizumab Experimental group (221 patients): high-dose vitamin C plus FOLFOX+/−bevacizumab	Experimental group was treated with high-dose vitamin C (1.5 g/kg/d intravenously for 3 h from day 1 to day 3) plus mFOLFOX6 with or without bevacizumab Treatment was continued for a maximum of 12 cycles or until disease progression, unacceptable toxicities, or a decision by the physician or patient to withdraw from the trial	High-dose vitamin C plus chemotherapy failed to show superior progression-free survival and overall survival compared with chemotherapy in patients with advanced CRC. Vitamin C significantly increased progression-free survival in *RAS*-mutated cancers compared to *RAS* wild-type tumors (9.2 months versus 7.8 months, 95% CI 0.5–0.91, *p* = 0.01).	[99]
Retrospective study	Total: 180 patients Control: 150 patients Vitamin C: 30 patients	Vitamin C 1 g/kg iv twice weekly for more than 5 weeks	Vitamin-C-treated patients had a lower recurrence and mortality rate, however, this was not statistically significant.	[104]
Retrospective observational study	CRC samples (50 patients)	Measurements of intratumoral vitamin C level, HIF1α, BNIP3 and VEGF expression Patient survival was monitored for 6 years after surgery	Inverse relationship between tumor vitamin C level and HIF1 pathway activation as well as tumor size. High tumor vitamin C level is associated with longer disease-free survival in the first 6 years after surgery.	[47]
Clinical intervention study	Total: 15 colon cancer patients Control (6 patients): without vitamin C Experimental group (9 patients): vitamin C substitution	Patients with colon cancer were randomized to receive infusions of up to 1 g/kg ascorbate for 4 days before surgical resection (9 patients) or to not receive infusions (6 patients)	While the trial was not powered to determine clinical efficacy, patients were followed up for the first 30 days and then up to two years postsurgery. Patients in the control cohort tended to have a longer hospital stay postsurgery. Ascorbate levels increased in all tumor regions, suggesting that increased plasma availability ensured effective accumulation throughout the tumor. Glut1 expression, but not VEGF or CA-IX expressions, all markers of HIF1 pathway activation, was significantly lower in tumors treated with vitamin C compared to untreated tumors.	[105]
Prospective observational study	Inclusion of 711,891 individuals from the Cancer Prevention Study-II Nutrition Cohort cancer-free in 1982 and followed for 14 years	Vitamin C use was determined by questionnaires	Use of vitamin C was not associated with CRC mortality. Subgroup analyses showed vitamin C supplementation for >10 years was associated with reduced risk of CRC mortality before age 65 and reduced risk of rectal cancer mortality at any age.	[107]
Prospective observational study	Inclusion of individuals from the Cancer Prevention Study-II Nutrition Cohort cancer-free in 1992/1993 and diagnosed with CRC through 2015	Vitamin C supplements were self-reported on questionnaires at baseline, in 1997, and every 2 years thereafter Pre- and postdiagnosis data were available for 3176 and 2006 CRC survivors, respectively, among whom 2116 (648 with CRC) and 1256 (242 with CRC) died	Multivitamin use before or after diagnosis is not associated with mortality in CRC survivors.	[108]
Prospective cohort study	2096 CRC cases with 703 cases of *KRAS* and *BRAF* mutation Average follow-up 12.0 years	Vitamin C intake 400 mg/day orally	Evidence for a potential benefit of vitamin C for CRC patients with *KRAS*- or *BRAF*-mutated tumors.	[109]
Phase II single-arm, multicohort clinical study	Study in progress NCT04033107 Enrollment of 30 patients with advanced hepatocellular carcinoma, advanced pancreatic cancer, advanced gastric cancer and advanced CRC who failed standard therapies	Participants will receive iv vitamin C injection (dose: 1.5 g/kg, days 1–3, every 2 weeks) with metformin 4 g daily orally, treatment termination when the disease progression is confirmed	Primary endpoint: progression-free survival.	[100]
Phase III randomized controlled study	Study in progress NCT04516681 Enrollment of 400 patients with advanced CRC expressing high levels of GLUT3	Experimental group: combined vitamin C (1.5 g/kg/day, days 1–3) every 2 weeks with adebrelimab and chemotherapy FOLFOX ± bevacizumab Control group: chemotherapy alone FOLFOX ± bevacizumab	Primary endpoint: objective response rate: CT or MRI scans to assess overall tumor response rate (complete and partial response). Secondary endpoints: progression-free survival, overall survival.	[101]
Phase II	Study in progress NCT04801511 Enrollment of 60 patients with locally advanced rectal cancer	High-dose iv vitamin C (24 g/day) will be delivered on the day of intensity-modulated radiation therapy (IMRT) followed by 5–6 cycles of mFOLFOX6 chemotherapy and radical surgery 10–12 weeks after IMRT	Primary endpoint: pathological complete remission. Secondary endpoints: acute toxicity, resection rate of anus, 2-year survival rate, 2-year disease-free survival.	[102]
Phase Ib	Study in progress NCT06336902 Enrollment of 15 patients with *KRAS*-mutant metastatic CRC	Patients receive botensilimab iv on day 1 of each cycle for up to 4 cycles Patients receive balstilimab iv and vitamin C iv on days 1, 15 and 29 of each cycle Patients undergo a fasting-mimicking diet on days −4 to −1 of each cycle Cycles repeat every 42 days for up to 2 years in the absence of disease progression or unacceptable toxicity	Primary endpoint: proportion of patients who adhere to the fasting-mimicking diet. Incidence of adverse events. Secondary endpoints: overall response rate, progression-free survival, overall survival.	[103]
Prospective clinical trial	Patients with terminal cancer diagnosis Total: 39 CRC: 9	Vitamin C 10 g iv twice with a 3-day interval and oral intake of 4 g vitamin C daily for a week Quality of life assessed with EORTC QLQ-C30	Patients report significantly fewer symptoms and increased scores for cognitive and physical functions.	[111]
Randomized controlled double-blind study	Total: 100 patients with colon cancer treated by colectomy Experimental group (49 patients): vitamin C Control group (48 patients): placebo	50 mg/kg vitamin C or placebo iv immediately after induction of anesthesia Morphine consumption and pain scores assessed at 2, 6, and 24 h after completion of surgery	High-dose vitamin C infusion decreased postoperative pain during the first 24 h and reduced morphine consumption in the early postoperative period.	[112]
Prospective cohort study	Patients with metastatic CRC (46 patients), cancer-free control group (45 patients)	Sociodemographic, lifestyle, medical variables, *BRAF* and *KRAS* mutations, as well as vitamin C plasma level and food intake	mCRC patients have lower plasma vitamin C levels than healthy controls. The trend toward higher mortality in the vitamin-C-deficient cancer group was not statistically significant.	[113]

## Data Availability

No new data were created or analyzed in this study. Data sharing is not applicable to this article.

## Abbreviations

The following abbreviations are used in this manuscript:

CRC	Colorectal cancer
DHA	Dehydroascorbic acid
SVCTs	Sodium-dependent vitamin C transporters
ROS	Reactive oxygen species
NAC	N-acetylcysteine
NAD	Nicotinamide adenine dinucleotide
H_2_O_2_	Hydrogen peroxide
TCA	Tricarboxylic acid
ICT	Immune checkpoint therapy
TET	Ten-eleven translocation
